# School-based health centers as an approach to address health disparities among rural youth: A study protocol for a multilevel research framework

**DOI:** 10.1371/journal.pone.0303660

**Published:** 2024-05-15

**Authors:** Xue Zhang, Mildred E. Warner, Sharon Tennyson, Wendy Brunner, Elaine Wethington, John W. Sipple

**Affiliations:** 1 Department of City and Regional Planning, Cornell University, Ithaca, NY, United States of America; 2 Department of Global Development, Cornell University, Ithaca, NY, United States of America; 3 Jeb E. Brooks School of Public Policy and Department of Economics, Cornell University, Ithaca, NY, United States of America; 4 Bassett Research Institute, Center for Rural Community Health, Bassett Medical Center, Cooperstown, NY, United States of America; 5 Department of Sociology and Department of Psychology, Cornell University, Ithaca, NY, United States of America; PLOS: Public Library of Science, UNITED KINGDOM

## Abstract

School-Based Health Centers (SBHCs) are important healthcare providers for children in medically underserved communities. While most existing research on SBHCs has focused on urban environments, this study protocol proposes a mixed-methods, multi-level research framework to evaluate the role of SBHCs in addressing health disparities among underserved children and adolescents in rural communities. The study area includes four high-poverty rural counties in New York State served by Bassett Healthcare Network that permits a comparison of school districts with SBHCs to those without SBHCs, all served by providers within the Bassett Healthcare Network. We employ a human ecological framework that integrates the micro layer of individuals and families, the meso layer of school districts and community institutions, and the macro layer of local and state policies. Our research framework first identifies the socioecological health risk factors, and then proposes innovative strategies to investigate how SBHCs impact them. We propose evaluating the impact of SBHCs on the individual (micro) level of child healthcare utilization using patient records data. At the meso level, we propose to investigate how School-SBHCs partnership may facilitate greater cross-agency collaboration and broader structural and social determinist of health to address health disparities. At the macro level, we propose to assess the impact of SBHCs and cross-agency collaboration on outcomes associated with a culture of community health. This study protocol will enable researchers to assess how SBHCs reduce rural health disparities, and provide evidence for organizational and public policy change.

## 1. Introduction

School-Based Health Centers (SBHCs) are authorized by federal and state legislation to expand access to healthcare for underserved children and adolescents by providing primary and preventive care in the school setting [1–3]. SBHCs are sponsored by healthcare systems or hospitals and are staffed by multi-disciplinary teams of providers in consultation with a supervising physician, and typically offer care at little to no cost to students [4]. SBHCs enhance healthcare access and reduce school absenteeism, which may improve health, educational outcomes, and individual and community wellbeing [5,6]. There are an estimated 3,900 SBHCs in the U.S., with 36% of SBHCs in rural schools. However, with important exceptions [7–10], most research has focused on urban environments. A Community Guide systematic review points to a research gap in SBHC effectiveness in “rural areas with low population density.”[3]

Rural residents face health disparities, including higher mortality rates [11,12], and socioecological risk factors that adversely impact health, such as high levels of family poverty and disruption of family structures [13–16]. Children living in rural communities have higher rates of exposures to Adverse Childhood Experiences, which are associated with poor health outcomes in adulthood [17]. Many rural communities in the U.S. are healthcare “deserts” without health service providers [18], and have limited public services and infrastructure [14,16,19] and built environments that do not promote public health [20,21].

Collaboration is important in rural areas facing both service deficits and health disparities [13,22,23]. Cross-agency collaboration is used not just for service delivery, but also for information and trust building [24,25]. Building trust can also promote bonding and bridging networks to encourage more participation of families with children and help build a more empowering approach for the schools and community agencies that serve them [19,21,26–29]. A collaborative community response is needed to build a culture of health [30,31], and is key to building healthy places for children and families [24,26,27,32–34].

Schools are a key partner in cross-agency collaborations to deliver services in rural communities [26,32,35], and provide measurable economic and social benefits to the communities they serve [36–39]. In recognition of these linkages, we propose that School-Based Health Centers (SBHCs) may be an effective cross-agency collaboration in addressing rural health disparities. Our study protocol evaluates, at multiple levels, how a network of SBHCs offering comprehensive healthcare in a disadvantaged rural region addresses health disparities among underserved rural youth. We employ a human ecological framework that integrates the micro layer of individuals and families, the meso layer of healthcare and school institutions, and the macro layer informing local and state policy.

Multilevel research on reducing health disparities is often limited by methodological, data, and assessment challenges [40]. The proposed research protocol leverages a unique study setting to combine quantitative analysis of patient healthcare visits from electronic health record (EHR) data and mixed methods analysis of cross-agency collaboration to demonstrate the effects of a network of rural SBHCs and identify integrated service provision and partnerships that have the potential to reduce health disparities in poor and underserved rural communities. We will use qualitative (semi-structured interviews and focus groups) and quantitative (survey, administrative, and EHR) data to address the following aims:

- Assess the impact of SBHCs on healthcare utilization by children in rural communities.
- Assess the impact of SBHCs on cross-agency collaboration contributing to child health and wellbeing in rural communities.
- Use a community-based participatory research (CBPR) framework [41,42] to identify key community, organizational and relational processes through which SBHCs link to communities.
- Assess whether SBHCs and cross-agency collaboration are associated with improved school attendance and increased healthcare utilization for family and community members beyond those enrolled in the SBHCs.

## 2. Conceptual model

**Fig 1:**
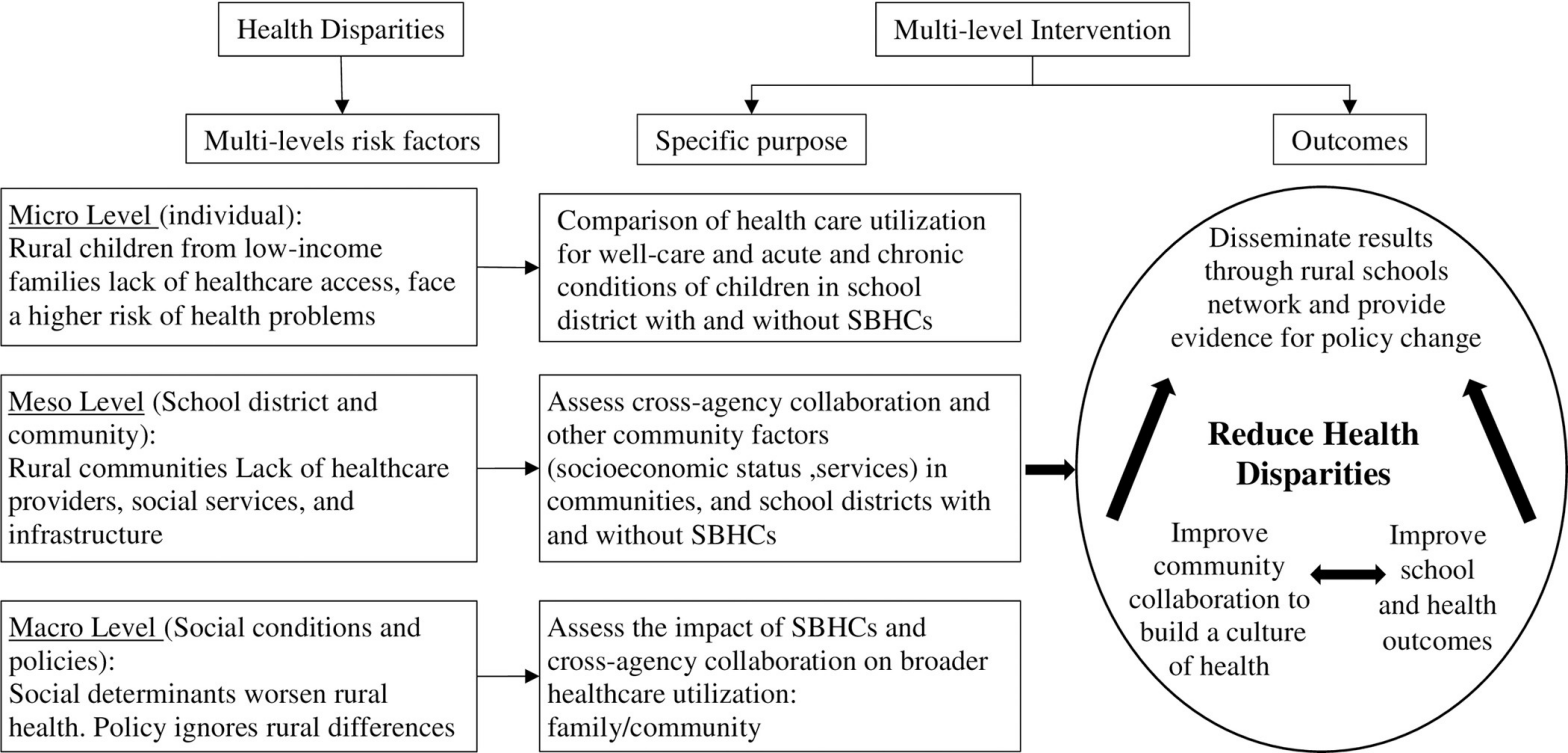
The conceptual framework of implementing multilevel human ecological logic model to address rural health disparities.

Fig 1 shows the conceptual framework of this research protocol. Community social conditions and higher-level policies contribute to the fundamental causes of health disparities [40,43–46], creating socioecological risk factors at the individual, institutional, community, and social conditions and policy levels in rural communities. As discussed in the introduction, at the individual (micro level), rural children from low-income families lack healthcare access, and face a higher risk of health problems [11,13–17]. At the institution and community (meso) level, rural communities lack healthcare providers, social services, and infrastructures [14,16,18,19,21,32]. At the social conditions and policy level (macro), social determinants worsen rural health [18], and policy may not be attuned to rural differences [21,23]. These conditions imply that successful implementation of efforts designed to reduce health disparities requires a long-term multilevel approach [40,47–49]. The dynamic collaboration between different entities is underdeveloped in public health research [50,51], but research shows these multiple levels of influence play an important role in improving population health [21,32,51].

SBHCs offer high-quality, comprehensive, patient-centered care and provide children with a medical home; this improves individual health but also helps create a culture of health by building trust and strengthening community relationships [52]. Establishing and maintaining SBHCs requires a multi-agency partnership and extensive collaboration among healthcare providers, schools, families and local institutes. Through these mechanisms, we hypothesize a positive effect of SBHCs on child health and wellbeing and on broader community health and wellbeing. This study protocol uses a multilevel framework to investigate these connections in order to identify effects of SBHCs on individual health and wellbeing, community collaboration, and school attendance (Fig 1).

On the individual (micro) level, this study evaluates the impact of SBHCs on child healthcare utilization. On the school and community (meso) level, this study focuses on social service agencies. This study will assess whether school districts with SBHCs have more cross-agency collaboration. On the policy (macro) level, this study will integrate the multiple levels of analysis to assess the relationship between SBHCs and changes in school attendance, and healthcare utilization for patients outside of school attendance age groups. This will enable us to test whether SBHC’s can improve school outcomes and reduce community health disparities and will provide evidence for organizational and public policy change.

## 3. Materials and methods

### 3.1 Study area

**Fig 2:**
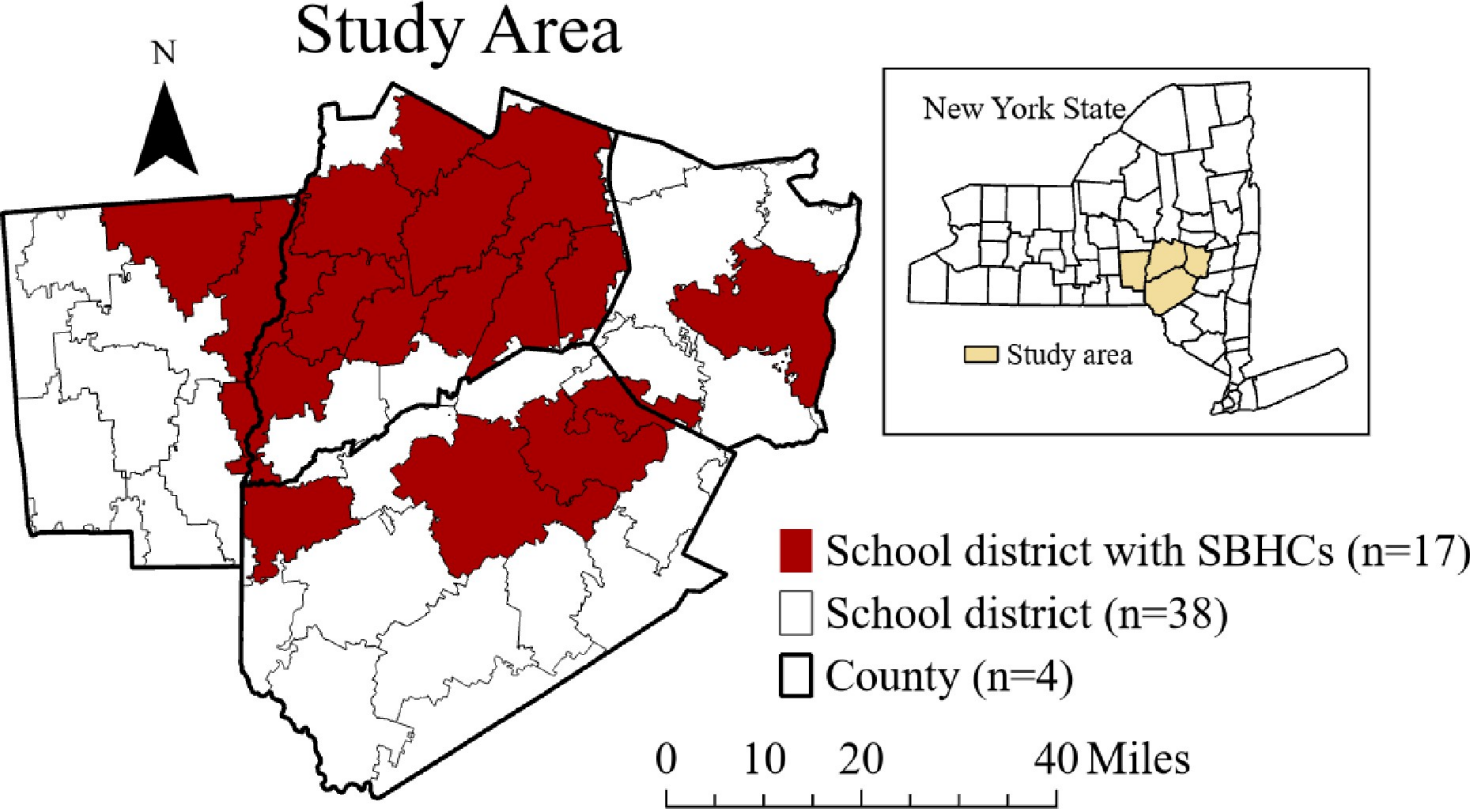
Study area.

This study evaluates the impact of SBHCs on individual and community health in four distressed rural counties of New York State. Bassett Healthcare Network operates 21 SBHCs in 17 of 38 school districts in four rural counties. Bassett is the main healthcare provider in the region, operating a network of primary and specialty care clinics, emergency departments and hospitals in the four-county region (Fig 2).

Table 1 shows the opening dates, 2023 enrollment rates, and SBHC locations. The first School-Based Health Center was established in 1992. Most recently, the 22^nd^ site was opened in the 4^th^ quarter of 2023. Because of the rurality, most school districts in this region have only one school at each level (elementary, middle, high school) and in many, these are located on a single campus. Hence, the SBHCs offer healthcare to all students in the district. On average, mature SBHCs serve more than 80% of students in their schools.

**Table 1:** Characteristics of bassett healthcare network’s SBHCs.

Open year	School district	County	2023% SBHC enrollment	# sites
1992	Delhi Central School District	Delaware	81%	2
1996	Edmeston Central School District	Otsego	98%	1
1996	Laurens Central School District	Otsego	80%	1
1996	Morris Central School District	Otsego	75%	1
2002	South Kortright Central School District	Delaware	82%	1
2003	Sherburne-Earlville Central School District	Chenango	90%	2
2007	Cooperstown Central School District	Otsego	86%	2
2007	Middleburgh Central School District	Schoharie	74%	2
2007	Worcester Central School District	Otsego	85%	1
2010	Milford Central School District	Otsego	73%	1
2010	Schenevus Central School District	Otsego	73%	1
2010	Sidney Central School District	Delaware	79%	1
2010	Stamford Central School District	Delaware	84%	1
2012	Unadilla Valley Central School District	Chenango	85%	1
2015	Richfield Springs Central School District	Otsego	76%	1
2020	Gilbertsville-Mount Upton Central School District	Otsego	81%	1
2023	Cherry Valley-Springfield Central School District	Otsego	-	1

Data source: Authors collection.

Bassett SBHCs provide comprehensive healthcare, preventive dental care and mental health services with no out-of-pocket costs to families [2]. They provide referrals to specialists and connect patients with community resources [53]. Services are available in the summer and over school holidays. Bassett’s SBHCs have received National Center for Quality Assurance (NCQA) recognition as Patient Centered Medical Homes (PCMH). Patient and parent surveys and communications with local school superintendents show that parents and children trust the SBHCs [5].

Bassett opens SBHC sites after working extensively with local school and community partners and meeting with important constituent groups (students, parents, school health staff, administrators and teachers, Board of Education, and local officials). Historical records on SBHC openings and discussions with Bassett administrators suggest that factors including physical space, funding, and Bassett personnel availability are key determinants of SBHC siting [54].

### 3.2 Data collection and analytic strategy

Based on the conceptual framework (Fig 1) this study protocol examines the role of SBHCs in addressing rural health disparities at the micro level (individual), meso level (school district and community), and macro level (social conditions and policies).

#### 3.2.1 Micro level (individual)

At the micro level, this study protocol will assess the impact of SBHCs on healthcare utilization among school-aged children. In a systematic review of the literature on SBHC to promote health equity, Knopf et al. [3] identify specific factors in a unifying human ecological framework through which SBHCs improve educational and health outcomes leading to health equity for low-income and minority students. SBHCs provide health education, increased access to healthcare services and increased use of preventive care to student patients. In providing services, SBHCs build relationships with students, increasing students’ comfort with obtaining healthcare. This feeds back into increased use of services, leading to increased and earlier diagnoses of acute and chronic conditions and decreased risk behavior. Ultimately, access to comprehensive and timely healthcare through SBHCs results in decreased morbidity, decreased misuse of medical care, and improved health and educational outcomes. The research literature on SBHCs in urban areas shows that SBHCs are associated with an increased use of preventive healthcare [55] and dental care [56], improved asthma care resulting in decreases in asthma hospitalizations [57–59], and decreased frequency of depressive episodes and suicide ideation [60]. We hypothesize that the SBHCs in our rural study area will also provide direct health benefits and will substantially increase access to routine, preventive, and chronic disease care.

We will assemble a 12-year (2011–2022) panel dataset including de-identified patient visit data extracted from Bassett’s electronic health record (EHR) system, patient household socioeconomic (SES) data, and school district data. We will estimate student household SES by linking patient address to the validated Housing-based Index of Socioeconomic Status (HOUSES) index which is based on publicly available property data. School district SES data will be collected from the American Community Survey (ACS) and the New York State Education Data & Research Hub (NYEdData).

The patient data include visits to any Bassett healthcare facility and distinguishes patient visits to an SBHC from visits to another Bassett facility. Analyses will focus on comparing Bassett pediatric patients with access to SBHCs and those without. Because the database is de-identified, we cannot directly observe whether a patient is enrolled in an SBHC. One method to deal with this is to assume that all students living in a school district with an SBHC are enrolled in that SBHC (i.e., intent to treat analysis, or ‘whole school effects’) [3,10]. A second (individual-level) method is to categorize students as “enrolled in the SBHC” if they have at least one SBHC visit on record in the database within a year and compare patients utilizing an SBHC to students in districts without SBHCs. These approaches reduce issues of bias associated with patient selection into SBHC enrollment.

Cross-sectional and longitudinal data on patient healthcare visits in SBHC and non-SBHC school districts facilitates rigorous analysis of research questions that could not be studied with cross-sectional or payer data alone, including comparison of trends and levels of healthcare utilization over time, with a separate focus on the usage of telemedicine visits during the COVID-19 pandemic; comparison of chronic disease management in relation to recommended guidelines [61]; and examining the impact of SBHC openings and associated changes in healthcare utilization.

#### 3.2.2 Meso level (school district and community)

At the meso level, this study protocol will assess the impact of SBHCs on cross-agency collaboration. Low-income families face a patchwork of potential support systems, and in underserved rural communities these services can be difficult to access. Increasingly, hospitals and healthcare systems are screening for and addressing social determinants of health (e.g. food insecurity, housing) in the clinical setting [62]. In our dynamic, human ecological framework (Fig 1), the meso layer of community institutions reaches up to the macro layer for policy change and down to the micro system layer to promote inclusion of families with children. This, in turn, builds engagement and trust of families in the service system. Cross-agency collaboration is an integral aspect to building a community response [23,24,32], and a key factor to improve social and health service provision [35,63]. We hypothesize that districts with SBHCs will have a higher level of cross-agency collaboration.

Using a mixed methods approach, we will collect data on the extent of cross-agency collaboration among healthcare providers and community organizations at the county level and across school districts. We will use interviews and focus groups with key informants and community agencies to gather our community partners’ insights on community features that lead to cross-agency collaboration, especially the role of SBHCs. These interviews and focus groups with key informants will provide insights to design the questions for a larger set of focus groups with community agencies. Consent forms will be sent to participants prior to interviews and focus groups, and verbal consent will be obtained before the sessions. The community agency focus groups will identify key issues of interest to our partners, explore barriers and strategies to collaboration and inter-organizational trust in rural communities and motivators for program change. We will use factors identified in the qualitative research to design a survey to send to school superintendents and community partners in the four-county study region. The survey will explore the role of the school and community service agencies in connecting across agencies (school, county government, nonprofit) to provide a full range of services for children and their families (e.g., nutrition, health, youth services, recreation, housing, transportation), types of partnerships, forms of service delivery, levels of collaboration, and barriers and motivators for collaboration at the county and school district level.

Mixed methods will be used to analyze the data on the role of SBHCs in building collaboration at the county and school district level. Drawing from collective impact theory [64] and collaborative governance theory [65], we will use thematic coding to measure five features of cross-agency collaboration at the county level: common agenda, shared measurement of program outcomes, mutually reinforcing activities, communication mechanisms, and the role of backbone agency support. We will ensure inter-rater reliability through our coding method. We will use member checking and participant review to ensure the quality of the interviews and focus groups with informants and services agencies, and use both code saturation and meaning saturation to ensure we conduct enough interviews and focus groups. Our survey data will be used to create indices that measure cross-agency collaboration for each school district and county. The indices will measure cross-agency collaboration, including the services provided through collaboration, the types of partnerships, barriers, and motivations. These measures will allow us to compare school districts with and without a SBHC. We will examine the collaborative networks among community agencies to describe, explore, and understand the relational and structural aspects of community agency networks to support public health. This analysis also will enable us to explore connections between agencies (health, education, social service, libraries) and the role of different levels in the system (county government, school district).

#### 3.2.3 Macro level (social conditions and policies)

This study protocol will assess the impact of a collaborative SBHC network on school attendance and broader family and community access to healthcare. Our analysis is founded in a human ecological systems approach, in which the micro, meso and macro system levels each have important and interlinked influences on outcomes (Fig 1). We posit that, by establishing a more horizontal and participatory system of healthcare that builds bonding and bridging networks, SBHCs build community social capital [5,28,66], which in turn promotes behaviors that improve individual and community health outcomes [67,68]. Integrating the consolidated framework for implementation research (CFIR) [69] with our multilevel analysis, the study will analyze how individual, institution, and community forces combine to address health disparities.

We hypothesize that SBHCs will lead to higher rates of school attendance and greater healthcare utilization among family and community members beyond those enrolled in the SBHC, and that higher levels of cross-agency collaboration will independently and in combination with SBHCs positively impact healthcare utilization among family and community members. School attendance will be assessed using individual level data on school attendance from schools in our four-county study region across multiple years. The dataset will be compiled by the regional Board of Cooperative Educational Services (BOCES) district office and provided to us in de-identified form. To examine family and community healthcare utilization we will expand analysis of the Bassett EHR database to include the youngest children (ages 0 to 3), young adults (ages 21–25), and older adult patients (ages 25 and over). These analyses will incorporate indicators of community cross-agency collaboration obtained from the meso level analysis, to assess the relationships between SBHC exposure and cross-agency collaboration, community context, and education and community healthcare utilization. Although the cross-agency collaboration data are cross-sectional, community collaborations evolve slowly over time. Our analysis will use cross-sectional EHR data from only the last 4 sample years (2018–2022) to more closely match the time period in which data on collaborations are collected.

### 3.3 Results dissemination

We will use the community-based participatory research framework [41,42] and the Consensus Conference Model [70] to guide research, data integration and dissemination of results with community and policy stakeholders. We will bring together stakeholders to comment collectively on research questions and strategies and policy recommendations that emerge from the multilevel data analyses. We will develop research-based findings papers written at a level accessible to stakeholders, convene a conference of stakeholders, research experts, and community members, and discuss activities designed to elicit feedback and recommendations from those convened [41,42,70]. Applying this framework, we will identify opportunities to promote practice changes, expanded community partnerships, and new public policies.

Our multilevel analysis of a rural SBHC network, and our focus on how community, institutional and environment level modifiers are linked to the impact of SBHCs on care utilization and health and education outcomes will provide new insights into the impact of the SBHC intervention in rural communities and will yield generalizable conclusions of use to medically underserved rural communities in helping to understand the dynamics of community responses to rural health.

## 4. Discussion

Implementation research demonstrates the importance of developing an evidence base for successful programming [47,48]. Our project will enhance understanding of the impact of SBHCs in rural communities by identifying the critical factors at multiple levels that affect service provision and will help inform replication efforts in rural areas across the nation. While SBHCs are a known effective intervention, our study protocol allows us to address important limitations in existing literature evaluating SBHC interventions [71], which must contend with service differences across SBHCs [3], face challenges of limited enrollment of students into SBHCs [72] and limited statistical power [71], and have access to only short follow-up times or cross-sectional data [73]. Additionally, we can address gaps in SBHC research on how community, institutional and environment level modifiers are linked to the effectiveness of SBHCs, and the impact of SBHCs on families and community wellbeing. We will employ the Consolidated Framework for Implementation Research [69] to identify factors shaping multilevel interventions which impact health outcomes and disparities in rural communities.

The National Institute on Minority Health and Health Disparities (NIMHD) research framework includes five domains of influence on health disparities at four levels of health outcomes–the individual, family, community, and population [49]. This is based on a human ecological framework, but the interaction of institutional, community, and policy factors affecting health disparities are less developed aspects of the NIMHD framework [40,46,49]. Our proposed research incorporates a focus on these latter factors to study community level institutional collaboration and broader community level health outcomes associated with rural SBHCs. The proposed project will have transformative impact by focusing directly on the importance of these factors in reducing health disparities in poor and underserved rural communities.

By utilizing a unique panel dataset of all patient encounters (with SBHCs, community clinics, doctor offices, regional hospitals) over 12 years, our research will strengthen existing evidence on SBHCs using cross-sectional and longitudinal analysis of healthcare access and treatment in a rural healthcare network. Because Bassett Healthcare Network is the primary healthcare provider in our study region, their EHR database includes a substantial majority of the universe of people living in the region. EHR records are linked over time by a unique patient ID which allows for comparison of children “growing up” and attending school in a school district with an SBHC or in a school district without an SBHC. It allows for monitoring of diagnoses at a young age and follow-up treatment through high school in a school district with a SBHC or not. It allows observation of community members outside of school attendance age groups and how their health behaviors are similar or different across those living in school districts with and without SBHCs.

By producing evidence on cross-agency collaborations in rural communities with SBHCs, our project will bring innovative insights on how SBHCs may address social determinants of health and improve community health outcomes. By integrating analysis at the individual, school district, and community level, we will assess the role of SBHCs in improving school and community health outcomes. We will use results in partnership with an Advisory Board of school, community, and state policy representatives to provide evidence for practice and policy change to enhance effectiveness of SBHCs in promoting health equity in underserved rural areas.

### 4.1 Limitations

In analyzing the impact of SBHCs, a key potential bias arises due to nonrandom assignment of SBHCs to school districts. Preliminary analysis suggests that this is not a major source of concern in our setting [5], but we will have ample data on quantitative and qualitative differences across school districts to examine, with the objective of reducing, potential biases due to such nonrandom assignment. A second concern is the potential for selection effects in patient enrollment in SBHCs. The high fraction of students in each district who enroll in the SBHCs, Bassett’s predominance in the local healthcare market, the relatively homogeneous patient population, and our access to records of patient visits to all Bassett healthcare facilities (not just the SBHCs) reduce these concerns in our study setting. We will nonetheless use a variety of statistical approaches to reduce potential bias in our estimates. A third limiting factor is structural changes in access to health insurance due to Medicaid expansion implemented January 2014 in NYS. We deal with this problem by setting 2014 as the start date for longitudinal analyses of healthcare utilization. COVID-19 introduced a fourth potential limitation in generalizing findings from that period. Our individual patient record analysis will be conducted separately for the pre-COVID period through 2019 vs the COVID period (2020+) to allow for examination of COVID disruptions and changes in service models and healthcare utilization.

We also acknowledge that external validity related to different but equally homogenous communities or to diverse rural communities must be considered. Our study population is similar to many rural communities, especially in the northern and central regions of the U.S. and shares key challenges of declining economies and access to health care with many others. While we recognize that results may not be fully generalizable, our multilevel research approach will help inform the generalizability of our findings to other communities by providing evidence on community, institutional and environmental factors impacting the effects and effectiveness of the SBHCs.

## 5. Conclusion

Our project will employ a dynamic human ecological framework in which community institutions like SBHCs address health disparities at the individual level and facilitate local collaboration to promote a culture of community health (Fig 1). In this study protocol, we connect those layers through analysis of 1) individual patient records, differentiated by school districts with and without SBHCs, 2) cross-agency collaboration to promote the effectiveness of the community to advance health equity, and 3) broader community outcomes data on school performance and healthcare utilization in families and communities. We will utilize the community-based participatory research framework [41,42] and Consolidated Framework for Implementation Research [69] to investigate the multilevel connections between individuals, school districts, and community institutions, and how SBHCs impact them. The analytical approach addresses potential confounders such as selection of sites for SBHCs, patient selection into SBHC enrollment, limitations of patient visit data, and variations in SES measures across school districts. The study setting (a homogeneous rural area with limited patient migration, a SBHC network with uniform service provision, a near-universe of patient records, and time-varying introduction of SBHCs) reduces threats to internal validity that arise in other settings. The qualitative research component and local data on schools and communities will help mitigate other study design limitations. Results will enhance understanding of how SBHCs in medically underserved rural communities may shape health outcomes, impact community networks, and contribute to individual and community wellbeing, and will identify critical factors to inform rural health policy.
